# Impact of Age on Long-Term Outcomes of Laparoscopic Nissen Fundoplication—A Single Center Study

**DOI:** 10.3390/medicina60050688

**Published:** 2024-04-24

**Authors:** Natalia Dowgiałło-Gornowicz, Justyna Kacperczyk, Anna Masiewicz, Karolina Osowiecka, Paweł Lech

**Affiliations:** 1 Department of General, Minimally Invasive and Elderly Surgery, Collegium Medicum, University of Warmia and Mazury, Niepodległosci 44 St., 10-045 Olsztyn, Poland; lechpawel@op.pl; 2 Department of Anesthesiology and Intensive Care, Children’s Memorial Health Institute, Av. Dzieci Polskich 20, 04-730 Warsaw, Poland; justyna.kacperczyk@gmail.com; 3 Department of Neurology, Military Institute of Medicine, Szaserów 128 St., 04-141 Warszawa, Poland; annalukuc@gmail.com; 4 Department of Psychology and Sociology of Health and Public Health, School of Public Health, University of Warmia and Mazury in Olsztyn, Warszawska 30 St., 11-041 Olsztyn, Poland; k.osowiecka86@gmail.com

**Keywords:** elderly, gastroesophageal reflux disease, GERD, Nissen fundoplication, laparoscopic Nissen fundoplication

## Abstract

*Background and objectives:* Gastroesophageal reflux disease (GERD) is a common disease affecting approximately 20% of the adult population. This study aimed to compare the results of laparoscopic Nissen fundoplication (LNF) in the treatment of GERD in patients of different age groups. *Materials and Methods:* A retrospective analysis was performed on patients who underwent LNF in one surgical department between 2014 and 2018. Patients were divided into three groups based on age: under 40 years of age, 40–65 years of age, and over 65 years of age. *Results:* A total of 111 patients (44.1% women) were analyzed in this study. The mean age was 50.2 ±15 years, and the mean follow-up was 50 months ± 16.6 months. Recurrence of symptoms occurred in 23%, 20%, and 23% in each age group, respectively (*p* = 0.13), and 85%, 89%, and 80% of patients from the respective groups reported that they would recommend the surgery to their relatives (*p* = 0.66). Furthermore, 83%, 92%, and 73% of patients from the respective age groups reported that they would undergo the surgery again with the knowledge they now had (*p* = 0.16). *Conclusions:* Given these results and observations, LNF has been shown to be a good method of treatment for GERD in every age group. In our study, there were no differences found in terms of satisfaction with surgery and associated recommendations between the studied age groups.

## 1. Introduction

Gastroesophageal reflux disease (GERD) is a common disease affecting approximately 20% of the adult population [1,2]. It has its roots in the regurgitation of gastric contents into the esophagus, leading to several troublesome symptoms, categorized as typical, atypical, and extraesophageal. The main symptom of GERD is heartburn, defined as a burning sensation behind the sternum caused by the activation of chemoreceptors in the esophageal mucosa. Another common symptom is acid reflux, which is characterized by a feeling of acidic stomach contents in the mouth or lower part of the esophagus [1,2,3]. Atypical symptoms include epigastric pain, a feeling of fullness, and nausea [4]. Extraesophageal symptoms, such as chronic cough or chronic laryngitis, may seem less specific, but pose a significant problem for patients [1,5]. It should be emphasized that untreated GERD leads to Barrett’s esophagus, a metaplasia of the esophageal epithelium that induces predisposition to esophageal cancer with a high mortality rate [6]. All of these symptoms significantly impair functioning and make the daily lives of patients miserable. The symptoms can be truly disruptive, reducing quality of life and preventing work or other daily activities [7,8]. Patients often seek help from multiple specialists before a diagnosis is made, especially if the symptoms are non-specific. This means that the disease often becomes chronic, and patients suffer from symptoms for a long time, eventually requiring special help from physicians [9]. If the diagnosis is made, GERD is usually treated with proton pump inhibitors (PPIs), leading to rapid relief of symptoms [10,11]. However, after discontinuing PPIs, symptoms return in 90% of cases, even after just a few days. The administration of PPIs may be associated with side effects such as diarrhea and headaches. Moreover, especially in older adults, a risk of deficiencies of certain vitamins and minerals, including vitamin B12, has been associated with the long-term use of PPIs [12]. Finally, as many as 30% of patients do not respond to PPI therapy, and as such, it is important to explore other potential ways to treat GERD patients. Therefore, administering medications alone is not the only solution for relieving GERD symptoms. Patients diagnosed with GERD may be offered surgical treatment, which is also effective in patients who respond to PPI therapy. Moreover, surgical procedures can cure up to 93% of patients [3]. One of the most commonly performed methods is laparoscopic Nissen fundoplication (LNF). This is a safe and effective anti-reflux procedure [1,11] which involves creating a wrap from the fundus of the stomach around the distal part of the esophagus. Thus, the pressure in this part of the esophagus increases, which increases the lower esophageal sphincter.

According to the World Health Organization (WHO), by 2050, the percentage of the world’s population aged over 60 years will nearly double to 22% [13]. Contrary to popular belief, hydrochloric acid secretion does not decrease with age, and persists in over 80% of older patients. It is also known that reflux occurs more often in elderly patients compared to younger ones [14]. Due to the aging of society, more and more elderly patients with GERD symptoms who may qualify for surgical treatment are turning to surgical departments for assistance. However, there are still questions as to whether such patients should be operated on, and whether the benefits of surgery outweigh the possible perioperative risks [15,16].

This study aimed to compare the results of LNF treatment in patients of different age groups.

## 2. Materials and Methods

### 2.1. Patients

This study presents a retrospective analysis of patients who underwent LNF for the treatment of GERD in one surgical department between 2014 and 2018. All patients underwent preoperatively objective diagnosis of GERD according to the Lyon consensus [17]. The diagnosis included esophagogastroduodenoscopy and/or 24-h pH-monitoring. The inclusion criteria were an abnormal gastroscopy or pH-monitoring result, along with symptoms of GERD. In terms of gastroscopy, the diagnosis was confirmed with the occurrence of esophagitis LA grade B, C, or D, Barret’s esophagus, signs of hiatal hernia, or signs of cardiac insufficiency. In pH-monitoring, the occurrence of GERD was confirmed in cases of above 6% acid exposure time, or above 80 episodes of reflux. The exclusion criteria were refusal to take part in the study and follow-up, or failure to meet the criteria for a GERD diagnosis.

### 2.2. Study Design

Patients were divided into three groups depending on age: under 40 years of age, 40–65 years of age, and over 65 years of age. Before surgery, each patient answered questions from a provided survey. This survey contained demographic (age, gender) questions, and questions regarding symptoms according to the GERD-IS. Postoperative consultations were held one month after surgery, and then every twelve months after surgery. The consultation questionnaire included information on current symptoms of GERD according to the GERD-IS, the incidence of disease recurrence, and the need for PPI administration. Moreover, it consisted of two “yes/no” questions to assess satisfaction with the results of LNF surgery. The first question asked whether the patient would recommend the surgery to his or her relatives. The second question concerned whether the patient, having undergone the procedure, would choose to undergo the procedure again with their current knowledge. Not all of the patients provided clear answers to both of these questions, so the number of patients surveyed differed for each.

### 2.3. Study Outcomes

The primary endpoint was patient satisfaction after the procedure, expressed in the resolution of symptoms and recommendations for their relatives. The secondary endpoint was the occurrence of major complications according to the Clavien–Dindo classification, and recurrences after surgery [18].

### 2.4. Surgical Technique

**Figure 1:**
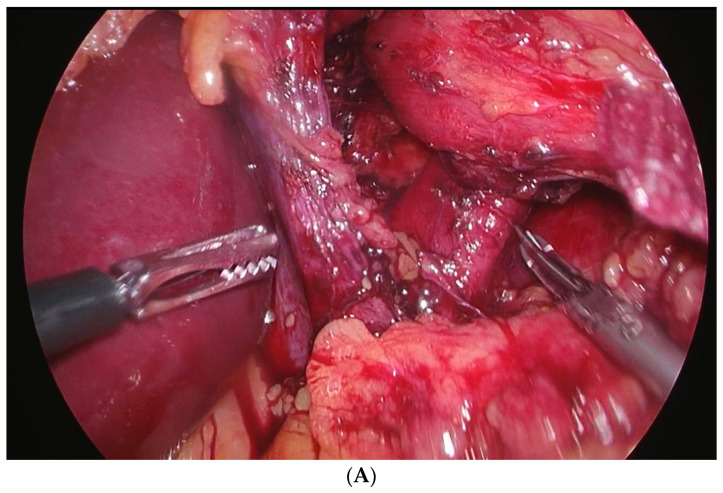

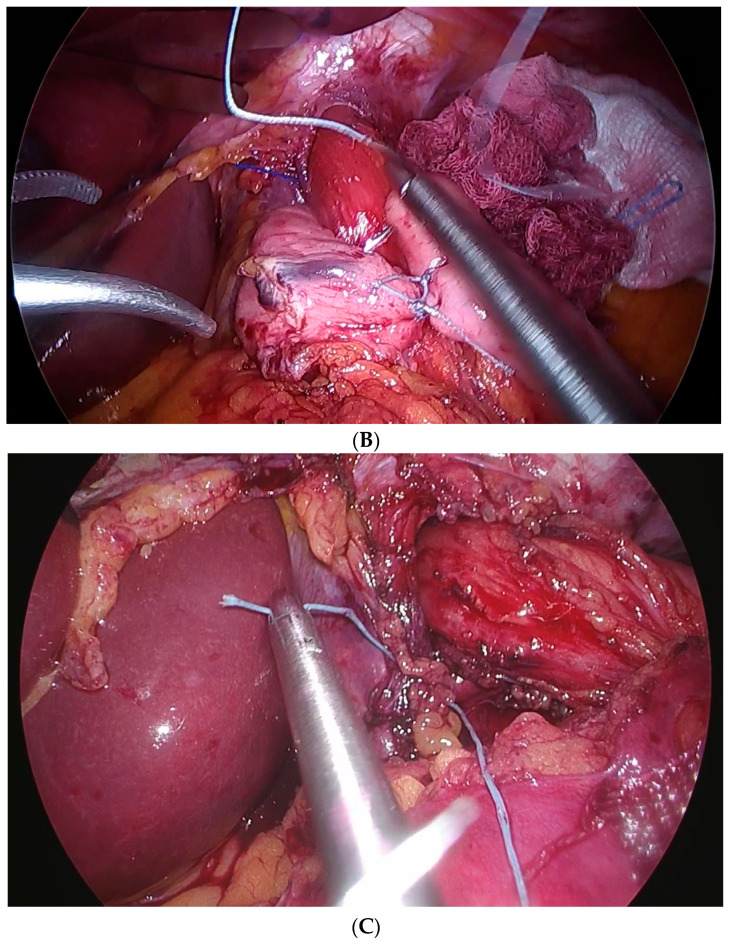
(**A**) Dissection of diaphragmatic crura; (**B**) Sewing diaphragmatic crura; (**C**) Performing wrap of the fundus.

All patients underwent LNF surgery according to a standard procedure. All of the LNF surgeries were performed by the same team of experienced surgeons. In the years considered by this study, the department performed over 50 LNFs per year, which represented one of the largest number of procedures in the country. The patient was placed supine on the operating table with the lower limbs abducted in the anti-Trendelenburg position. Carbon dioxide was insufflated into the peritoneal cavity to a pressure of 12 mm Hg. The first 10 mm trocar was inserted in the midline, approximately 12 cm below the xiphoid process, then under the eye, 10 mm in the right axillary line below the costal arch (liver retractor), 5 mm in the right midclavicular line (dissector), 10 mm in the left mid-clavicular line (Cordless Ultrasonic Dissection System, Sonicision™, Medtronic, Dublin, Ireland), and 5 mm in the left anterior axillary line under the left costal arch (assistant). The LNF started with the dissection of the crura of the diaphragm and then the content of the mediastinum, as seen Figure 1A. The distal esophagus was mobilized to a length of approximately 5 cm into the abdominal cavity. Then, the crura were sutured behind the esophagus with two or three nonabsorbable sutures (polyfilament, PremiCron^®^/Dagrofil^®^, size 0, BBraun, Spain), as seen Figure 1B. A posterior 360° fundus wrap of 3 cm in length was performed using two or three nonabsorbable sutures (polyfilament, PremiCron^®^/Dagrofil^®^, size 2/0, BBraun, Spain), as seen in Figure 1C. A 36F bougie was inserted to check for any restrictions or difficulties in passage. Usually, no drains were left.

### 2.5. Follow-Up

Patients were mobilized and allowed to drink water orally 2 h after surgery. Ondansetron was administered as an antiemetic, and metamizole/paracetamol as an analgesic as needed. Discharge home took place on the first postoperative day if patients reported no problems with swallowing and their general condition did not raise any concerns. A soft diet and PPIs (20 mg of omeprazole daily) were recommended for a month after the surgery. Patients were followed-up after one month, and then once every year after the surgery. During the study period, 135 laparoscopic LNFs were performed in our department, of which 24 patients were lost to follow-up. The follow-up rate was therefore 82.2%.

### 2.6. Statistical Analysis

A descriptive statistical analysis was conducted. All data were analyzed using Statistica software 13 PL (StatSoft Inc., Tulsa, OK, USA). Quantitative variables were characterized by mean and standard deviation, whereas qualitative variables were presented using percentages. To check whether a quantitative variable came from a normally distributed population analysis, the Shapiro–Wilk W test was used. The median, first quartile (Q1), and third quartile (Q3) of the GERD-IS distribution were assessed. Differences in GERD-IS before and after the surgery were assessed using the Wilcoxon test. To compare medians for various factors, the Mann–Whitney test was performed. The chi-square test was used to compare proportions in the analyzed subgroups. A *p*-value of <0.05 was considered statistically significant.

## 3. Results

A total of 111 patients (49 women, 44.1%) were analyzed in this study. The mean age was 50.2 ± 15 years, and the mean follow-up was 50.3 ± 16.6 months (Table 1). Patients were divided into three groups depending on age. There were 48 patients under 40 years of age, 41 patients between 40 and 65 years of age, and 22 patients over 65 years of age.

A total of 17 patients (15.3%) required chronic PPI administration after the surgery. They did not differ significantly in age; however, in patients over 40 years of age, the percentage of PPIs administered was slightly higher (*p* = 0.19) (Table 2). Recurrence of symptoms occurred in 23% of patients under 40 years of age, 20% of patients aged 40 to 65 years, and 23% of patients over 65 years of age (*p* = 0.92),Table 2. According to the Clavien–Dindo scale, there were no statistically significant differences in complications of grade III severity or higher between each age group (*p* = 0.13). In the youngest group, there were no early postoperative complications. There were three early postoperative complications of grade IIIb, according to Clavien–Dindo classification, in patients aged 40–65. In one patient, the spleen was iatrogenically injured intraoperatively, and it was decided that it should be removed. Two patients required re-operation within the first 30 days after surgery. The patient exhibited dysphagia due to the narrowing of the wrap, and problems with swallowing. The wrap was resewn, and a good postoperative outcome was then achieved. One patient had early recurrence of symptoms; it was found that there was a failure in the wrap, and it was performed again during reoperation. In the oldest group, two patients had complications. One patient had a grade IVa complication; this was acute respiratory failure just after the surgery, without a previous history of respiratory distress. Conservative treatment was introduced for this patient, with a good outcome. One other patient had a grade IIIb complication. This patient required a reoperation due to dysphagia; the fundoplication wrap turned out to be too tight and was resewn. There was no mortality across any of the age groups.

The patients answered two “yes” or “no” questions. The first question was whether they would recommend LNF to their relatives. The lowest percentage (80%) of patients who answered positively was in the oldest group, and the highest was in the middle group (89%), but the “yes” response rate did not differ significantly between the groups (*p* = 0.66) (Table 2). In regard to the question of whether they would undergo the surgery again with the knowledge they have now, the lowest percentage of positive answers was in the oldest group (73%). The differences between the groups were not statistically significant (*p* = 0.16) (Table 2).

All of the patients filled out the GERD-IS questionnaire. A statistically significant improvement in symptoms was observed for each of the nine GERD-IS questions in different age groups, but there were no statistical differences between age groups (Table 3).

## 4. Discussion

The global population is aging every year, and more and more patients over 65 years of age appear in our daily practice. Problems arise when such patients, previously called “elderly”, are assessed for qualification for surgical treatment of a disease whose symptoms can be regulated by drugs. However, as research has shown, drugs only alleviate the symptoms, and the disease continues to develop [11,19]. This study demonstrated the impact of age on patients’ outcomes of LNF surgery in long-term follow-up. There was a slight trend towards higher satisfaction rates in middle-aged patients (40–65 years), and the lowest satisfaction rates were observed in those over 65 years of age, but this result was not found to be statistically significant. Our analysis showed that patients over 65 years of age can achieve similar benefits to younger patients after undergoing LNF surgery, while also maintaining a similar risk of complications.

Pizza et al. presented one of the first papers on the effectiveness of LNF by comparing younger and older patients [20]. The authors studied 338 patients under 65 years of age and 65 patients over 65 years of age. According to the study, excellent LNF results were achieved by 92.9% of younger and 91.9% of older patients, with the same risk of complications observed across both groups. Similar observations were described by Cowgill et al. [21]; in their study, 81% of patients under 60 years of age and 82% of patients over 70 years of age experienced symptom relief after LNF. Beck et al. also confirmed that age did not affect anti-reflux fundoplication results, with LNF representing about 85% of the analyzed surgeries [22]. This is consistent with our results.

Other authors have reported conflicting results. Maret-Ouda et al. demonstrated that age over 60 years, compared to age under 45 years, was associated with an increased risk of the recurrence of symptoms, with an adjusted hazard ratio of 1.41 (95% CI, 1.10–1.81) [23]. Their paper included a large sample (*n* = 2655), but it did not differentiate the type of surgery, and the outcomes were based solely on the use of PPIs, so this may have introduced some bias. Moreover, this study did not analyze additional factors apart from age, such as the presence of comorbidities. We can assume that the worse results in elderly patients could have resulted from their multi-morbidities and changes spreading throughout the body, including frailty syndrome [24]. Song et al. investigated the connection between muscle strength in older adults and GERD [25]. The authors demonstrated that muscle strength was found to be independently and inversely associated with GERD. Therefore, it can be assumed that the older the patient, the weaker the muscular elements of the anti-reflux barrier in the body are, and thus, recurrences are more likely to manifest over the years.

Zhou et al. published an interesting study on the reoperation rate after anti-reflux surgery [26]. Their analysis showed that the reoperation rate was significantly higher in younger patients (HR = 3.56 for 30 years of age; HR = 1.89 for 30–50 years of age; HR = 1.65 for 50–65 years of age). This differs from the previous study, and the reason for this remains unclear. However, the authors suggested that younger patients were more likely to be candidates for revisional surgery than older patients, due to their better conditions of health and longer life expectancy. Moreover, this paper had some limitations: the type of surgery analyzed was unknown, and both laparoscopic and open procedures were analyzed.

There has been much debate lately about whether age should be a patient eligibility rule. Addo et al. analyzed not only operating outcomes, but also quality of life, after anti-reflux surgery in different age groups [16]. They demonstrated that older patients were at higher risk of complications or reoperations, but that there were no differences in quality of life after the surgery. They and other authors noted that the frequency of complications was associated with a higher incidence of comorbidities and a higher ASA score, which is not only related to age [16,27,28,29]. It seems that when qualifying a patient for surgery, we should consider their biological age, not their chronological age. A patient’s comorbidities and general condition may predispose them to an increased risk of complications.

Finally, it is necessary to mention the recent review of The Society of American Gastrointestinal and Endoscopic Surgeons (SAGES) regarding GERD management [30]. Based on an assessment of 444 patients who underwent any anti-reflux surgery, no differences were observed between patients under and over 65 years of age. Patients over 65 years of age also reported a significantly lower rate of recurrence of heartburn compared to younger patients (5.8% vs. 26.3%, *p* < 0.001). This again allows us to put forward the thesis that anti-reflux surgeries should not be contraindicated in patients over 65 years of age, due to their effectiveness and safety.

This study had some limitations. Despite this, it is worth noting that the study reports a long follow-up, and a relatively high follow-up rate for such a period of time. Unfortunately, after the first mandatory consultation in our center, some patients refused further consultations without complaining about any problems, which may indicate a lack of complications or a recurrence of symptoms, but unfortunately, we do not have this data. The main limitation is the retrospective nature of the study and the recruitment of patients from a single center. The number of patients is limited, but the study population corresponds to operations performed in one of the leading upper gastrointestinal surgery centers in the country. For this reason, we believe that our results can be applied to a broad population. We also did not have data on the individual results of preoperative imaging tests, such as gastroscopy or pH-monitoring, nor the duration of the disease or the duration of preoperative PPIs use. Moreover, we did not have the results of the objective control tests such as gastroscopy or pH-monitoring, and the test results were based on the subjective feelings of patients and the GERD-IS scale. Due to the reduced quality of life caused by GERD and the spectacular improvements observed after surgery, patients who get rid of their symptoms after surgery often refuse to undergo follow-up tests, reporting only if the symptoms return. In our study, however, we wanted to demonstrate patient satisfaction with the procedure, so we analyzed the disappearance of subjective symptoms.

The analysis contained in our publication is important and relevant. The group of patients is homogeneous; all patients had the same operation performed by one team in one center. The LNF surgical technique has been standard for years. The analysis is also based on long-term observations, with an average follow-up time of approximately 50 months. We believe that this study may be useful in making decisions about surgery in patients over 65 years of age.

## 5. Conclusions

LNF is a good method for treatment of GERD in every age group. According to our study, 80% of patients older than 65 years of age reported satisfaction with the surgery, and 73% of them reported that they would suggest the surgery to their relatives, and that with their current knowledge, they would undergo the surgery again. These results did not differ significantly from the results achieved in other age groups. Therefore, when qualifying patients for surgical anti-reflux treatment, we should not disqualify patients solely because of their older age.

## Figures and Tables

**Table 1:** Patients’ characteristics.

Variable	Value
Age, years (range, SD)	50.2 (18–80, ±15)
Gender, women/men, %	49/62 (44.1%)
Follow-up, months	50.0 (21.2–76.3, ±16.6)
PPIs after surgery, %	17 (15.3%)
Recurrence, %	24 (21.6%)

SD: standard deviation, PPIs: proton pump inhibitors.

**Table 2:** Patient characteristics and answers depending on age.

Variable	Age						
	<40 Years		40–65 Years		>65 Years		
	N	%	N	%	N	%	*p*
Recurrence	11	23%	8	20%	5	23%	0.92
Complications	0	0%	3	7%	2	9%	0.13
PPIs after surgery	4	8%	9	22%	4	18%	0.19
Recommendation of surgery for relatives	39	85%	32	89%	16	80%	0.66
Undergoing surgery again with current knowledge	39	83%	33	92%	16	73%	0.16

**Table 3:** Median and interquartile range (IQR) of GERD-IS before and after the surgery (1—daily; 2—often; 3—sometimes; 4—never).

How Often…	<40 Years		40–65 Years		>65 Years		*p*
	Median	25–75% IQR	Median	25–75% IQR	Median	25–75% IQR	
have you had pain in your chest or behind the breastbone?	4	3–4	4	3–4	4	3–4	0.49
have you had a burning sensation in your chest or behind the breastbone?	4	3–4	4	3–4	4	3–4	0.99
have you had regurgitation or an acid taste in your mouth?	4	4–4	4	3–4	4	3–4	0.06
have you had pain or burning in your upper stomach?	4	4–4	4	3–4	4	4–4	0.25
have you had a sore throat or hoarseness that is related to your heartburn or acid reflux?	4	4–4	4	3–4	4	4–4	0.15
have you had difficulty getting a good night’s sleep because of your symptoms?	4	4–4	4	3.5–4	4	4–4	0.16
have your symptoms prevented you from eating or drinking any of the foods you like?	4	3–4	4	3–4	4	3–4	0.33
have your symptoms kept you from being fully productive in your job or daily activities?	4	4–4	4	4–4	4	3–4	0.31
do you take additional medication other than what the physician told you to take (such as Maalox, Alusal, Manti)?	4	4–4	4	4–4	4	3–4	0.32

## Data Availability

The data presented in this study are available on request from the corresponding authors.
